# Measurable residual mutated *IDH1* before allogeneic transplant for acute myeloid leukemia

**DOI:** 10.1038/s41409-024-02447-4

**Published:** 2024-11-06

**Authors:** Gege Gui, Niveditha Ravindra, Pranay S. Hegde, Georgia Andrew, Devdeep Mukherjee, Zoë Wong, Jeffery J. Auletta, Firas El Chaer, Evan C. Chen, Yi-Bin Chen, Adam Corner, Steven M. Devine, Sunil G. Iyer, Antonio Martin Jimenez Jimenez, Marcos J. G. De Lima, Mark R. Litzow, Partow Kebriaei, Wael Saber, Stephen R. Spellman, Scott L. Zeger, Kristin M. Page, Laura W. Dillon, Christopher S. Hourigan

**Affiliations:** 1 Fralin Biomedical Research Institute, Virginia Tech FBRI Cancer Research Center, Washington, DC USA; 2 Department of Biostatistics, Johns Hopkins Bloomberg School of Public Health, Baltimore, MD USA; 3 Laboratory of Myeloid Malignancies, Hematology Branch, National Heart, Lung, and Blood Institute, National Institutes of Health, Bethesda, MD USA; 4 Center for International Blood and Marrow Transplant Research, NMDP, Minneapolis, MN USA; 5 The Ohio State University College of Medicine, Columbus, OH USA; 6 University of Virginia, Charlottesville, VA USA; 7 Dana-Farber Cancer Institute, Boston, MA USA; 8 Massachusetts General Hospital, Boston, MA USA; 9 Bio-Rad Laboratories, Pleasanton, CA USA; 10 Columbia University Irving Medical Center, New York, NY USA; 11 Sylvester Comprehensive Cancer Center, Miami, FL USA; 12 Mayo Clinic, Rochester, MN USA; 13 The University of Texas MD Anderson Cancer Center, Houston, TX USA; 14 Center for International Blood and Marrow Transplant Research, Medical College of Wisconsin, Milwaukee, WI USA

**Keywords:** Translational research, Cancer genetics, Acute myeloid leukaemia

## Abstract

Measurable residual disease (MRD) in adults with acute myeloid leukemia (AML) in complete remission is an important prognostic marker, but detection methodology requires optimization. Persistence of mutated *NPM1* or *FLT3*-ITD in the blood of adult patients with AML in first complete remission (CR1) prior to allogeneic hematopoietic cell transplant (alloHCT) associates with increased relapse and death after transplant. The prognostic implications of persistence of other common AML-associated mutations, such as *IDH1*, at this treatment landmark however remain incompletely defined. We performed testing for residual *IDH1* variants (*IDH1*m) in pre-transplant CR1 blood of 148 adult patients undergoing alloHCT for *IDH1*-mutated AML at a CIBMTR reporting site between 2013 and 2019. No statistically significant post-transplant differences were observed between those testing *IDH1*m positive (*n* = 53, 36%) and negative pre-transplant (overall survival (OS): *p* = 0.4; relapse: *p* = 0.5). For patients with *IDH1* mutated AML co-mutated with *NPM1* and/or *FLT3*-ITD, only detection of persistent mutated *NPM1* and/or *FLT3*-ITD was associated with significantly higher rates of relapse (*p* = 0.01). These data, from the largest study to date, do not support the detection of *IDH1* mutation in CR1 blood prior to alloHCT as evidence of AML MRD for increased post-transplant relapse risk.

## Introduction

Detection of measurable residual disease (MRD) prior to allogeneic hematopoietic cell transplant (alloHCT) in patients with acute myeloid leukemia (AML) in remission is associated with increased relapse and worse survival after transplant [1–11]. It has been recently reported that detection by ultra-sensitive next-generation sequencing (NGS) of mutated *NPM1* or *FLT3* internal tandem duplication (*FLT3*-ITD) persistence in first complete remission (CR1) blood is strongly associated with increased relapse and death compared to testing negative [12–14]. The genomic etiology of AML is heterogenous and well-described [15] but the prognostic implication of detecting persistence of all common AML-associated mutations in cytomorphological complete remission remains incompletely defined. Variants in isocitrate dehydrogenase (*IDH*) genes are observed in around 20% of patients diagnosed with AML (~7% for *IDH1*), and clinical outcomes are known to differ due to co-occurrence of other mutations, *IDH* subtypes, treatment strategies, and patient factors [16–21]. Targeted therapy for *IDH1*-mutated AML is available, and an association between residual *IDH1* mutations and post-transplant clinical outcomes may help provide evidence regarding post-alloHCT maintenance approaches [22–25]. Although some studies have examined the relationship between residual mutated *IDH1* in CR and subsequent clinical outcomes [21, 26, 27], definitive nation-level standardized evidence to inform decision making for the clinical utility of *IDH1* as a target for AML MRD testing was not previously available.

## Methods

### Patient population

Adult patients (18 years or older) undergoing first alloHCT for *IDH1* mutated AML in CR1 at a Center for International Blood and Marrow Transplant Research (CIBMTR) reporting site in the United States between 2013 and 2019 who participated in the CIBMTR database (NCT01166009) and repository (NCT04920474) protocols, had an available remission blood sample collected within 100 days before alloHCT, and follow-up clinical data including relapse and survival were included in this study. All patients gave written informed consent in accordance with the Declaration of Helsinki.

Patient baseline characteristics included age, sex, race, hematopoietic cell transplant-specific comorbidity, Karnofsky performance status, secondary AML, European LeukemiaNet (ELN) 2017 risk group, baseline genetics before transplant (*IDH1, IDH2, KIT, NPM1, FLT3-TKD, FLT3*-ITD), conditioning regimen, graft type, donor group, anti-thymocyte globulin (ATG) usage, and site-reported flow cytometry MRD status.

### Targeted next-generation sequencing (NGS)

Genomic DNA was extracted from remission blood and residual variants identified as previously reported [12]. In short, ultrasensitive error-corrected NGS targeting mutational hotspot regions of the *IDH1*, *NPM1*, and *FLT3* genes was performed utilizing an automated workflow with pre- and post-PCR separation. The assay was validated to detect variants down to at least 0.1% variant allele fraction (VAF) by performing serial dilutions of AML patient DNA containing known *IDH1* R132C or R132H mutations into normal DNA at a range of 5–0.005%. NGS libraries were sequencing on a NovaSeq 6000 (Illumina) with unique dual indices. Bioinformatic pipelines were used to perform error-corrected variant calling followed by filtering to identify residual disease. Raw FASTQ files are the NCBI Sequence Read Archive (Accession: PRJNA834073 and PRJNA997373).

### Digital droplet PCR (ddPCR)

A subset of 51 *IDH1* variants identified by NGS were orthogonally validated using ddPCR on the Bio-Rad QX200 or QX600 system as described previously [12].

### Statistical analysis

Logistic regression was implemented for determining the variables related to NGS *IDH1* MRD status. OS and cumulative incidence of relapse were considered as the primary endpoints, and the follow-up time was collected with day of transplant as time 0. Median follow-up time was calculated for censored patients. Kaplan-Meier estimation and log-rank tests were used to calculate OS and relapse-free survival endpoints. Cox proportional hazards models were fitted, with Lasso penalty for variable selection, and the proportional hazards assumptions were validated. Fine-gray regression models were used to examine the cumulative incidence of relapse with non-relapse mortality (NRM) as a competing risk, and Bayesian information criterion was used for model selection. Potential interactions between mutation groups and clinical characteristics were tested.

## Results

**Fig. 1:**
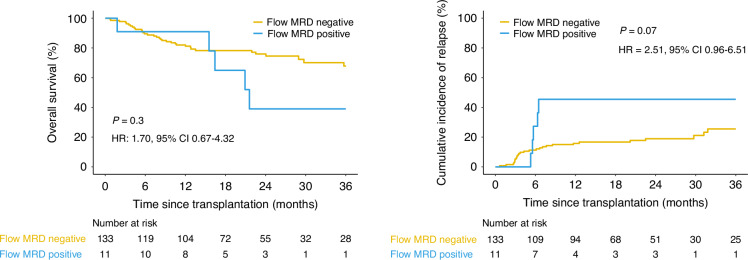
Clinical outcomes of flow cytometry MRD for *IDH1*-mutated AML patients after allogeneic hematopoietic cell transplant based on pre-transplant measurable residual disease (MRD) status. Overall survival (left) and cumulative incidence of relapse (with non-relapse mortality as competing risk; right) for patients with available flow cytometry MRD (*n* = 144) grouped by site-reported flow MRD positive (blue) or negative (yellow).

One hundred forty-eight patients with *IDH1* mutated AML and undergoing alloHCT in CR1 were included in the study, with a median follow-up time of 24 months. Relapses were reported in 37 patients (25%), the majority occurring within 1 year after alloHCT (*n* = 28, 76%; Supplementary Fig. 1). There were 46 death events reported and 27 (59%) were relapse related. Univariable competing risk regression analysis suggested that patients with intermediate (HR: 4.5, *p* = 0.04) or adverse (HR: 4.4, *p* = 0.05) ELN risk were more likely to relapse compared to those with favorable ELN risk (Table 1 and Supplementary Fig. 2). Pre-transplant remission flow cytometry results were reported to the CIBMTR by the transplant centers for 97% of the patients (*n* = 144) with a positivity rate of 8% (*n* = 11). Patients testing positive by flow cytometry pre-transplant had a trend of lower OS and higher cumulative incidence of relapse after alloHCT compared with those testing negative, but the results did not achieve statistical significance (OS: *p* = 0.3, HR = 1.70, 95% CI 0.67–4.32; relapse: *p* = 0.07, HR = 2.51, 95% CI 0.96–6.51; Fig. 1 and Supplementary Fig. 3).

**Table 1:** *IDH1*-mutated AML patient baseline clinical characteristics.

Variable	Number of patients (%)	Variable	Number of patients (%)
**Age**		**HCT-comorbidity index**	
<60	73 (49%)	0	35 (24%)
≥60	75 (51%)	1–2	46 (31%)
**Sex**		>2	67 (45%)
Female	79 (53%)	**Karnofsky score**	
Male	69 (47%)	<90	68 (46%)
**Conditioning intensity**		≥90	79 (53%)
MAC	80 (54%)	**ATG usage**	
RIC/NMA	68 (46%)	No	124 (84%)
**Site-reported flow cytometry**		Yes	24 (16%)
Negative	133 (90%)	**Race**	
Positive	11 (7%)	Caucasian	125 (84%)
**Graft type**		Other	19 (13%)
Peripheral blood	111 (75%)	**ELN risk group**	
Cord blood	15 (10%)	Favorable	30 (20%)
Bone marrow	22 (15%)	Intermediate	68 (46%)
**Donor type**		Adverse	50 (34%)
HLA-identical sibling	18 (12%)	**AML group**	
Matched unrelated	96 (65%)	De novo	122 (82%)
Haploidentical related	10 (7%)	Therapy-related	9 (6%)
Cord blood	12 (8%)	Transformed MDS/MPN	17 (11%)
Mismatched	9 (6%)	**Baseline mutation**	
Multiple donors	3 (2%)	* NPM1* and/or *FLT3*-ITD	69 (47%)

*MAC* myeloablative conditioning, *RIC* reduced-intensity conditioning, *NMA* nonmyeloablative, *AML* acute myeloid leukemia, *HLA* human leukocyte antigen, *HCT* hematopoietic cell transplant, *ATG* antithymocyte globulin usage, *ELN* European LeukemiaNet, *MDS* myelodysplastic syndrome, *MPN* myeloproliferative neoplasm, *ITD* internal tandem duplication.

**Fig. 2:**
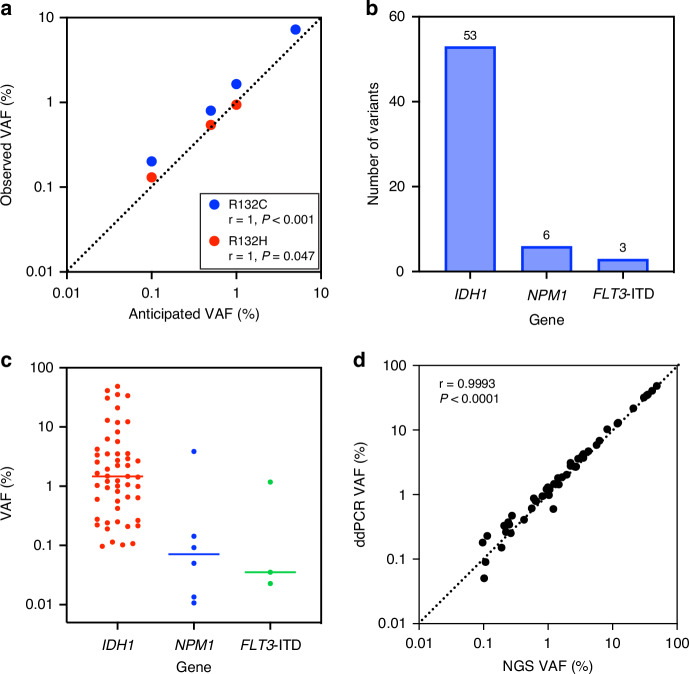
Detection of residual variants in pretransplant blood of *IDH1*-mutated AML patients during complete remission. **a** NGS MRD assay limit of detection for *IDH1* R132 variants was determined to be 0.1% variant allele fraction (VAF) by serial dilution of two *IDH1* mutations (R132C and R132H). The anticipated VAFs of *IDH1* variants were plotted versus the observed ones with a value of equivalence line displayed as a dotted line and the correlations for the two types respectively. **b** The total number and **c** VAF of variants per gene as detected by targeted next-generation sequencing (NGS) during remission prior to transplant in the peripheral blood of *IDH1-*mutated AML patients. **d** A total of 51 of the 53 detected variants in *IDH1* had an assay available for orthogonal validation by digital droplet PCR (ddPCR). The VAFs of *IDH1* variants detected by NGS (x-axis) were plotted versus ddPCR (y-axis), with a value of equivalence line displayed as a dotted line. A significant correlation was observed in the VAF as detected by NGS compared to ddPCR with an orthogonal validation rate of 100%. The Pearson correlation coefficient is shown on the inset of the graph.

**Fig. 3:**
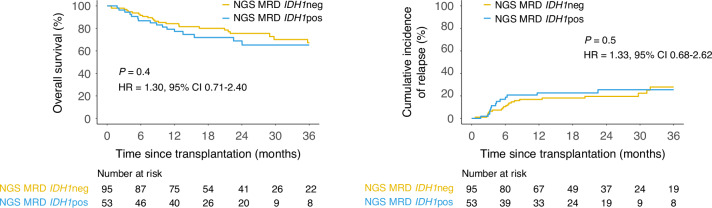
Clinical outcomes of *IDH1*-mutated AML patients after allogeneic hematopoietic cell transplant based on pre-transplant *IDH1* mutation persistence. Overall survival (left) and Cumulative Incidence of Relapse (with non-relapse mortality as competing risk; right) in patients with *IDH1*-mutated AML (*n* = 148) based on the presence (NGS MRD *IDH1*pos, blue) or absence (NGS MRD *IDH1*neg, yellow) of detectable residual *IDH1* variants in the blood pre-transplant during first complete remission.

A custom error-corrected NGS assay was used for detecting MRD and validated to detect *IDH1* variants down to a VAF of at least 0.1% (Fig. 2a). NGS MRD analysis identified residual *IDH1* mutations in the pretransplant remission blood of 53 (36%) patients at a median VAF of 1.5% (range 0.09–48.5%), with a 100% validation rate by digital droplet PCR for the variants with an orthogonal assay available (Supplementary Table 1 and Fig. 2b–d). Logistic regression indicated that *IDH1* NGS-MRD positivity was more likely in patients older than 40 years (OR: 1.02 with every 1-year increase after 40, *p* = 0.04). No statistically significant difference between the NGS-MRD *IDH1* positive and negative groups in clinical outcomes was detected (NGS MRD *IDH1* pos vs neg: OS HR 1.30, 95% CI 0.71–2.40, relapse HR 1.33, 95% CI 0.68–2.62; Fig. 3 and Supplementary Fig. 4a), which remained true after further subgroup analyses by age (Supplementary Fig. 4b) or when stratifying by high (≥2.5%) or low VAF groups (<2.5%, Supplementary Fig. 4c).

**Fig. 4:**
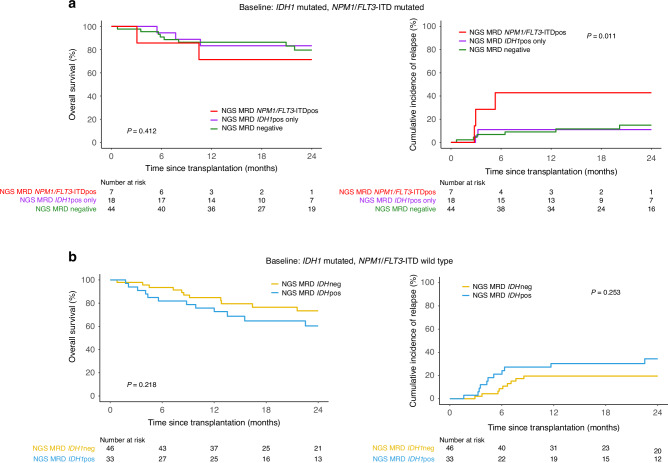
Clinical outcomes for *IDH1*-mutated AML patients after allogeneic hematopoietic cell transplant based on pre-transplant measurable residual disease (MRD) status stratified by co-mutated *NPM1* and/or *FLT3*-ITD at baseline. **a** Overall survival (OS; left) and cumulative incidence of relapse (CIR; with non-relapse mortality as competing risk; right) in patients with *IDH1*-mutated AML co-mutated with *NPM1* and/or *FLT3*-ITD (*n* = 69). Patients are stratified based on the presence of residual *NPM1* and/or *FLT3*-ITD variants regardless of residual *IDH1* (red, NGS-MRD *NPM1*/*FLT3*-ITDpos); the presence of residual *IDH1* variants in the absence of residual *NPM1*/*FLT3*-ITD (purple, NGS-MRD *IDH1*pos only); and the absence of residual *IDH1*, *NPM1*, or *FLT3*-ITD variants (green, NGS-MRD negative). **b** OS (left) and CIR (with non-relapse mortality as competing risk; right) in patients with *IDH1*-mutated AML without baseline *NPM1* and/or *FLT3*-ITD variants (*n* = 79). Patients are stratified based on the presence (NGS MRD *IDH1*pos, blue) or absence (NGS MRD *IDH1*neg, yellow) of detectable residual *IDH1* variants in the blood pre-transplant during CR1.

Patients with *IDH1-*mutated AML also often have mutations in *NPM1* and/or *FLT3*-ITD [15]. Our previous study focusing on patients with *NPM1* and/or *FLT3*-ITD mutated AML showed that patients with either of these mutations detected in CR1 prior to alloHCT had increased relapse and lower OS [12]. Within this cohort of *IDH1* mutated patients, we had information on the baseline mutational status of *NPM1* and *FLT3*-ITD, allowing for investigation into the impact of these co-mutations. A subset of 69 patients (47%) were co-mutated for *NPM1* and/or *FLT3*-ITD at baseline, and those with residual *NPM1* and/or *FLT3*-ITD mutations had higher rates of relapse compared to those with persistence of *IDH1* only or the NGS-MRD negative groups (*p* = 0.01, 43% vs 11% or 15% at 2 years, Fig. 4a and Supplementary Fig. 5a). The cohort excluding patients with baseline mutations *NPM1* and/or *FLT3*-ITD (*n* = 79, 53%) did not show any significant clinical outcome differences based on mutated *IDH1* persistence (Fig. 4b and Supplementary Fig. 5b).

We also examined the impact of the conditioning intensity regimens used for alloHCT in these patients and residual *IDH1* variants and saw no association with clinical outcomes (Supplementary Fig. 6). However, since the conditioning regimen was not randomly assigned, there were more older patients (age > 60 yrs) receiving reduced intensity/non-myeloablative conditioning compared with younger patients (71% vs. 21%; chi-square test: *p* < 0.001). Additionally, due to small sample size of the subgroups, there was insufficient number of patients to properly examine the interaction between age, conditioning intensity, and residual *IDH1* mutations.

Multivariable regression analyses for OS and relapse did not identify residual *IDH1* mutations as an important marker. ELN risk group remained to be significant for relapse (intermediate vs. favorable HR: 4.8, *p* = 0.04, 95% CI: 1.1–21), and the flow MRD positive group had higher risk for relapse compared to the negative group (HR: 2.6, *p* = 0.04, 95% CI: 1.1–6.7; Supplementary Fig. 7).

Ivosidenib, an *IDH1* inhibitor, was approved by the US FDA in July 2018 for use in patients with relapsed or refractory *IDH1*-mutated AML [28]. Since the registry data available for this patient cohort on the off-label use of medications as initial induction, pre-transplant “MRD-erasing” [29], or post-transplant “maintenance” [30] therapies is incomplete, the time period of this transplant cohort bridges this approval date and provides an opportunity to investigate this potential variable. No differences in NRM, relapse, relapse-free survival, or OS were detected when comparing patients transplanted either before or after July 2018 (Supplementary Fig. 8a), including when stratified by pre-transplant *IDH1* persistence status (Supplementary Fig. 8b).

## Discussion

The specific recurrent mutations commonly observed at initial AML diagnosis have been well described [15, 31, 32]. Routine profiling of these regions of interest using DNA-sequencing panels has allowed disease subclassification and prognostication. Evidence of MRD in remission has been associated with an increased risk of subsequent relapse and death [2, 33, 34], providing additional information beyond that offered by baseline genetic profiling at initial pre-treatment diagnosis. It is therefore tempting, but unproven, to propose screening for the persistence of all such mutations as an AML MRD test for patients in clinical complete remission.

The Pre-MEASURE project was designed to generate a robust evidence base, in a nation-wide retrospective cohort of adults with AML in first remission prior to first allogeneic transplant, of the actual prognostic implications of detecting specific persistent AML-associated mutations in pre-transplant blood [12–14, 35, 36]. The goal of this specific Pre-MEASURE sub-study was to determine for AML patients the association between pre-alloHCT persistent *IDH1* mutations and post-transplant clinical outcomes. We show that the detection of persistent *IDH1* mutations in the blood of adult patients with AML in CR1 prior to first alloHCT is common and not associated with increased relapse or death after transplant compared to those testing negative. For patients with *IDH1* mutated AML co-mutated with *NPM1* and/or *FLT3*-ITD, detection of persistent *NPM1* and/or *FLT3*-ITD was associated with higher rates of relapse. These data, from the largest study to date, do not support the detection of an isolated *IDH1* mutation in CR1 blood prior to alloHCT as evidence of increased post-transplant risk.

The finding that not all leukemia-associated mutations have prognostic significance when detected during remission in patients with AML has precedent [37, 38]. Isolated detection of mutations in other genes associated with DNA methylation (i.e., *DNMT3A, TET2*) is also not associated with increased relapse risk and should not be considered evidence of AML MRD. Suggestions regarding the mechanism underlying the observation reported here for *IDH1* would be speculative but include the possibilities that such mutations are from cells without the capacity to cause a leukemic relapse (e.g., either a non-malignant clonal hematopoietic precursor, an irrelevant cell lineage, and/or a differentiated cell type) or that an effective therapy prevented post-transplant relapse and death. For the latter possibility, it is not currently possible to quantify the antileukemic efficacy of alloHCT but one may explore the potential impact of targeted therapy. While our study had incomplete information on the specific treatment regimens used for these patients, including the usage of targeted agents, we did not see any differences in outcomes related to pre-transplant persistence of *IDH1* before or after the approval of Ivosidenib. Other medications, including venetoclax combinations, have been approved for patients with AML since the start of the cohort described here [39]. Additional studies will therefore be required to determine the specific implications of a persistent *IDH1* mutation, after a specific class of therapy, at a particular treatment timepoint. The need for more detailed information has motivated prospective clinical protocols, such as the MEASURE study (NCT05224661), collecting comprehensive clinical annotation on treatment regimens along with dense biobanking, including diagnostic and relapse samples. Additionally, randomized clinical trials will be required to demonstrate any benefit of targeted and/or other peri-transplant therapies to improve the outcomes of patients with *IDH1*-mutated AML undergoing alloHCT.

## Supplementary information

Supplementary Material

## Data Availability

Clinical data are/will be made publicly available from CIBMTR through their resource website https://cibmtr.org/CIBMTR/Resources/Publicly-Available-Datasets. Raw FASTQ files are at the NCBI Sequence Read Archive (SRA) (Accession: PRJNA834073 and PRJNA997373).
