# Supplementary material for: Measurable residual mutated IDH1 before allogeneic transplant for acute myeloid leukemia
Source: Bone Marrow Transplant. 2024 Nov 6;60(2):154–60. doi: 10.1038/s41409-024-02447-4 (PMC11810766; doi:10.1038/s41409-024-02447-4)
Supplement: Supplementary file 1 — Supplementary Material [file 41409_2024_2447_MOESM1_ESM.pdf]

Supplementary Data

Measurable Residual Mutated *IDH1* before Allogeneic Transplant for Acute Myeloid Leukemia

Gege Gui[1,2], Niveditha Ravindra[3], Pranay S. Hegde[3], Georgia Andrew[3], Devdeep Mukherjee[3], Zoë Wong[3], Jeffery J. Auletta[4,5], Firas El Chaer[6], Evan C. Chen[7], Yi-Bin Chen[8], Adam Corner[9], Steven M. Devine[4], Sunil G. Iyer[10], Antonio Martin Jimenez Jimenez[11], Marcos J.G. De Lima[5], Mark R. Litzow[12], Partow Kebriaei[13], Wael Saber[14], Stephen R. Spellman[4], Scott L. Zeger[2], Kristin M. Page[14], Laura W. Dillon[3]*, Christopher S. Hourigan[1]*
*LWD and CSH contributed equally to this work

[1]Fralin Biomedical Research Institute, Virginia Tech FBRI Cancer Research Center, Washington, DC
[2]Department of Biostatistics, Johns Hopkins Bloomberg School of Public Health, Baltimore, MD
[3]Laboratory of Myeloid Malignancies, Hematology Branch, National Heart, Lung, and Blood Institute, National Institutes of Health, Bethesda, MD
[4]Center for International Blood and Marrow Transplant Research, NMDP, Minneapolis, MN
[5]The Ohio State University College of Medicine, Columbus, OH
[6]University of Virginia, Charlottesville, VA
[7]Dana-Farber Cancer Institute, Boston, MA
[8]Massachusetts General Hospital, Boston, MA
[9]Bio-Rad Laboratories, Pleasanton, CA
[10]Columbia University Irving Medical Center, New York, NY
[11]Sylvester Comprehensive Cancer Center, Miami, FL
[12]Mayo Clinic, Rochester, MN
[13]The University of Texas MD Anderson Cancer Center, Houston, TX
[14]Center for International Blood and Marrow Transplant Research, Medical College of Wisconsin, Milwaukee, WI

Table of Contents

Supplementary Figure 1. Baseline characteristics for *IDH1*-mutated AML patients and the association with clinical outcomes after allogeneic hematopoietic cell transplant.	Page 2
Supplementary Figure 2. Univariable Cox regression for overall survival and relapse.	Page 3
Supplementary Figure 3. Site-reported flow cytometry MRD status for *IDH1*-mutated AML patients and the association with clinical outcomes after allogeneic hematopoietic cell transplant.	Page 4
Supplementary Figure 4. NGS MRD status for *IDH1*-mutated AML patients and the association with clinical outcomes after allogeneic hematopoietic cell transplant stratified by age and variant allele fraction.	Page 5
Supplementary Figure 5. NGS MRD status for *IDH1*-mutated AML patients and the association with clinical outcomes after allogeneic hematopoietic cell transplant stratified by baseline mutation groups.	Page 6
Supplementary Figure 6. NGS MRD status for *IDH1*-mutated AML patients and the association with clinical outcomes after allogeneic hematopoietic cell transplant stratified by conditioning intensity.	Page 7
Supplementary Figure 7. Multivariable regression analyses for the clinical outcomes of *IDH1*-mutated AML patients.	Page 8
Supplementary Figure 8. Patient transplant years for *IDH1*-mutated AML patients and the association with clinical outcomes after allogeneic hematopoietic cell transplant stratified by NGS MRD status.	Page 9
Supplementary Table 1. Variants detected by next-generation sequencing in the blood of *IDH1* mutated AML patients prior to transplant conditioning.	Page 10

Supplementary Figure 1. Baseline characteristics for *IDH1*-mutated AML patients and the association with clinical outcomes after allogeneic hematopoietic cell transplant. Cumulative incidence of non-relapse mortality (NRM, top left) and relapse (top right), relapse-free survival (RFS, bottom left) and overall survival (OS, bottom right) shown at 36 months based on baseline patient characteristics (A) overall and (B) by age group (<60yrs or ≥60yrs). Point estimates at different time points are shown in the table (far right). Overall P values: Gray's test for non-relapse mortality (NRM) and relapse; log-rank test for relapse-free survival (RFS) and overall survival (OS). P values for pointwise estimations at different time points: z-test. Confidence interval, CI; Probability, prob; Month, mo; Year, yr.

A

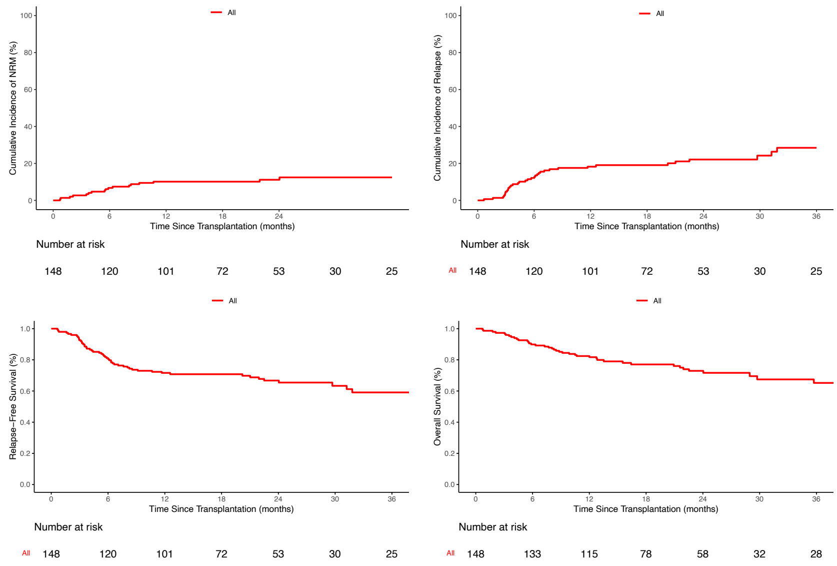

All patients

Outcomes	Prob (95% CI)
NRM	Overall n=148
3 mo	3(0-5)%
6 mo	7(3-11)%
1 yr	10(5-15)%
2 yr	11(6-16)%
3 yr	12(7-18)%
Relapse	Overall n=148
3 mo	5(1-8)%
6 mo	13(7-18)%
1 yr	19(13-25)%
2 yr	22(15-29)%
3 yr	28(19-37)%
RFS	Overall n=148
3 mo	93(87-96)%
6 mo	80(73-86)%
1 yr	71(63-78)%
2 yr	67(59-74)%
3 yr	60(50-69)%
OS	Overall n=148
3 mo	97(93-99)%
6 mo	90(84-94)%
1 yr	82(75-88)%
2 yr	73(64-80)%
3 yr	66(55-75)%

B

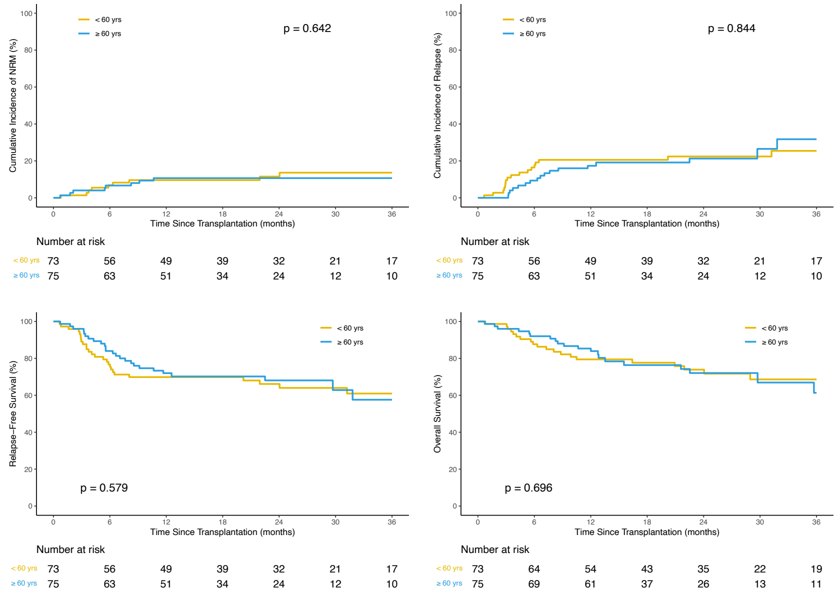

Age Group

Outcomes	Prob (95% CI)	Prob (95% CI)	p-value
NRM	<60yrs=73	≥60yrs=75	0.642
3 mo	1(0-4)%	4(0-8)%	
6 mo	7(1-13)%	7(1-12)%	
1 yr	10(3-16)%	11(4-18)%	
2 yr	11(4-19)%	11(4-18)%	
3 yr	14(5-22)%	11(4-18)%	
Relapse	<60yrs=73	≥60yrs=75	0.844
3 mo	10(3-16)%	0(0-0)%	
6 mo	16(8-25)%	9(3-16)%	
1 yr	21(11-30)%	17(9-26)%	
2 yr	22(13-32)%	21(11-31)%	
3 yr	25(14-37)%	32(15-48)%	
RFS	<60yrs=73	≥60yrs=75	0.579
3 mo	89(79-94)%	96(88-99)%	
6 mo	77(65-85)%	84(74-91)%	
1 yr	70(58-79)%	72(60-81)%	
2 yr	66(54-76)%	68(56-78)%	
3 yr	61(47-72)%	58(40-72)%	
OS	<60yrs=73	≥60yrs=75	0.696
3 mo	99(91-100)%	96(88-99)%	
6 mo	88(78-93)%	92(83-96)%	
1 yr	79(68-87)%	85(75-92)%	
2 yr	74(62-83)%	72(59-82)%	
3 yr	69(55-79)%	61(42-76)%	

Supplementary Figure 2. Univariable Cox regression for overall survival (A) and relapse (B). Baseline groups for the reported hazard ratio are 18-55yrs for age group, female for sex, MAC for conditioning intensity, negative for flow cytometry MRD, peripheral blood for graft type, matched unrelated for donor group, 0 for hematopoietic cell transplant specific comorbidity index (HCT-CI), <90 for Karnofsky score, no for ATG, white for race, favorable for ELN, no for *de novo*, negative for baseline *FLT3*-ITD, and negative for baseline *NPM1*.

	hazard ratio	p−value
Age: 55−65	0.868 (0.42−1.794)	0.702
Age: 65+	0.975 (0.486−1.957)	0.943
Sex: Male	1.431 (0.794−2.577)	0.233
Conditioning: RIC/NMA	0.87 (0.479−1.581)	0.647
Flow Cytometry MRD: Positive	1.695 (0.665−4.32)	0.269
Graft Type: Cord Blood	1.045 (0.43−2.544)	0.922
Graft Type: Bone Marrow	1.012 (0.446−2.299)	0.977
Donor Group: Other	1.425 (0.787−2.577)	0.242
HCT−CI: 1−2	1.517 (0.6−3.835)	0.379
HCT−CI: 3+	1.796 (0.772−4.178)	0.174
Karnofsky Score: 90+	0.662 (0.365−1.199)	0.173
ATG: Yes	0.894 (0.399−2.003)	0.785
race:Other	0.994 (0.433−2.282)	0.988
ELN: Adverse	2.188 (0.73−6.56)	0.162
ELN: Intermediate	2.48 (0.858−7.164)	0.093
Denovo: Yes	0.55 (0.282−1.071)	0.079
Baseline *FLT3*−ITD: Positive	0.851 (0.43−1.681)	0.641
Baseline *NPM1*: Positive	0.756 (0.394−1.452)	0.402

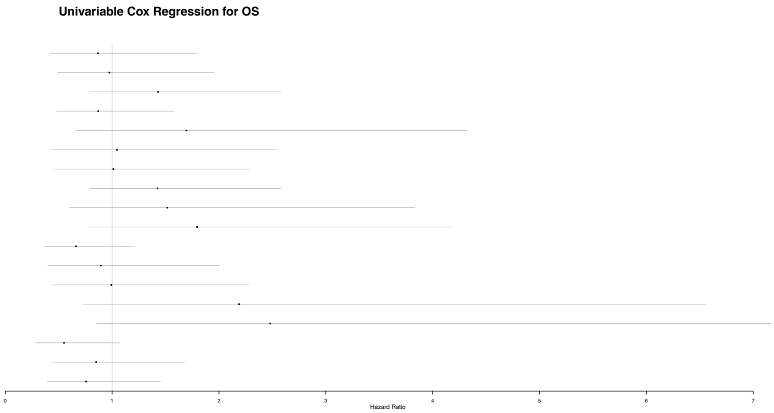

	hazard ratio	p−value
Age: 55−65	1.076 (0.484−2.392)	0.86
Age: 65+	1.187 (0.535−2.636)	0.67
Sex: Male	1.752 (0.914−3.356)	0.091
Conditioning: RIC/NMA	1.054 (0.554−2.005)	0.87
Flow Cytometry MRD: Positive	2.386 (0.927−6.138)	0.071
Graft Type: Cord Blood	0.56 (0.195−1.607)	0.28
Graft Type: Bone Marrow	1.006 (0.423−2.389)	0.99
Donor Group: Other	1.207 (0.634−2.299)	0.57
HCT−CI: 1−2	0.931 (0.34−2.546)	0.89
HCT−CI: 3+	1.472 (0.628−3.451)	0.37
Karnofsky Score: 90+	0.987 (0.525−1.854)	0.97
ATG: Yes	0.572 (0.21−1.559)	0.28
race:Other	0.869 (0.352−2.142)	0.76
ELN: Adverse	4.377 (1.02−18.785)	0.047
ELN: Intermediate	4.495 (1.084−18.64)	0.038
Denovo: Yes	0.673 (0.33−1.373)	0.28
Baseline *FLT3*−ITD: Positive	0.911 (0.428−1.937)	0.81
Baseline *NPM1*: Positive	0.548 (0.259−1.159)	0.12

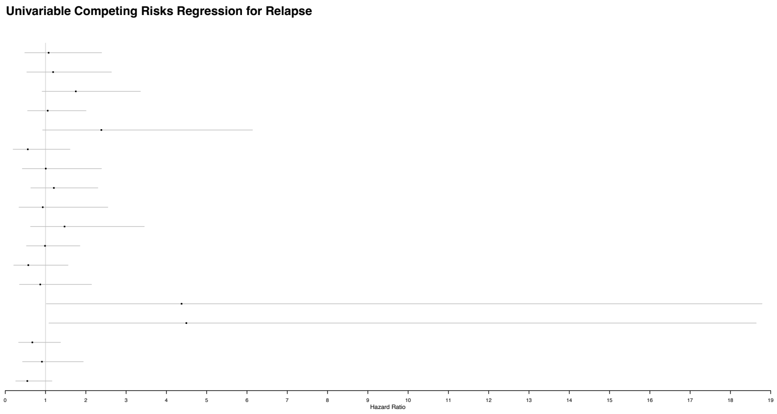

Supplementary Figure 3. Site-reported flow cytometry MRD status for *IDH1*-mutated AML patients and the association with clinical outcomes after allogeneic hematopoietic cell transplant. Cumulative incidence of non-relapse mortality (NRM, top left) and relapse (top right), relapse-free survival (RFS, bottom left) and overall survival (OS, bottom right) shown at 36 months for patients with data available for residual disease by flow cytometry as reported by the treatment site. Point estimates at different time points are shown in the table (far right). Overall P values: Gray's test for non-relapse mortality (NRM) and relapse; log-rank test for relapse-free survival (RFS) and overall survival (OS). P values for pointwise estimations at different time points: z-test. Confidence interval, CI; Probability, prob; Month, mo; Year, yr.

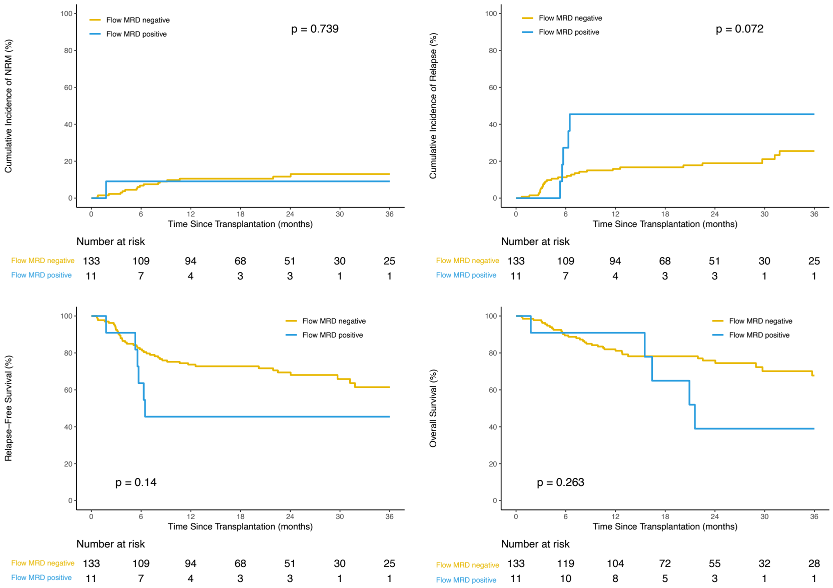

Site-Reported Flow Cytometry MRD

Outcomes	Prob (95% CI)	Prob (95% CI)	p-value
NRM	Flow MRD negative n =133	Flow MRD Positive n =11	0.739
3 mo	2(0-5)%	9(0-27)%	
6 mo	7(2-11)%	9(0-27)%	
1 yr	11(5-16)%	9(0-27)%	
2 yr	12(6-17)%	9(0-27)%	
3 yr	13(7-19)%	9(0-27)%	
Relapse	Flow MRD negative n =133	Flow MRD Positive n =11	0.072
3 mo	5(1-9)%	0(0-0)%	
6 mo	11(6-17)%	27(0-55)%	
1 yr	16(10-22)%	45(14-77)%	
2 yr	19(12-26)%	45(14-77)%	
3 yr	26(16-35)%	45(14-77)%	
RFS	Flow MRD negative n =133	Flow MRD Positive n =11	0.14
3 mo	92(86-96)%	91(51-99)%	
6 mo	82(74-88)%	64(30-85)%	
1 yr	74(65-80)%	45(17-71)%	
2 yr	69(60-77)%	45(17-71)%	
3 yr	61(50-71)%	45(17-71)%	
OS	Flow MRD negative n =133	Flow MRD Positive n =11	0.263
3 mo	98(93-99)%	91(51-99)%	
6 mo	89(83-94)%	91(51-99)%	
1 yr	82(74-88)%	91(51-99)%	
2 yr	76(67-83)%	39(9-69)%	
3 yr	68(56-77)%	39(9-69)%	

Supplementary Figure 4. NGS MRD status for *IDH1*-mutated AML patients and the association with clinical outcomes after allogeneic hematopoietic cell transplant stratified by age and variant allele fraction (VAF). Cumulative incidence of non-relapse mortality (NRM, top left) and relapse (top right), relapse-free survival (RFS, bottom left) and overall survival (OS, bottom right) shown at 36 months based on *IDH1* next generation sequencing measurable residual disease (NGS MRD) status for (A) all patients, (B) patients defined by age group (<60yrs or ≥60yrs), and (C) *IDH1* NGS MRD VAF groups (negative, 0%<VAF<2.5%, VAF≥2.5%). Point estimates at different time points are shown in the table (far right). Overall P values: Gray's test for non-relapse mortality (NRM) and relapse; log-rank test for relapse-free survival (RFS) and overall survival (OS). P values for pointwise estimations at different time points: z-test. Confidence interval, CI; Probability, prob; Month, mo; Year, yr; pos, positive; neg, negative.

A

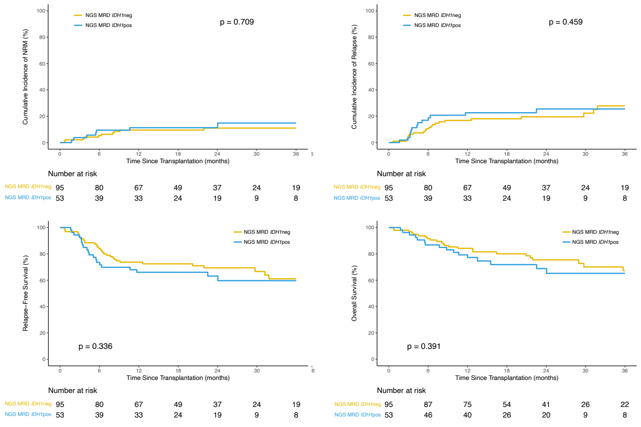

NGS MRD *IDH1*

Outcomes	Prob (95% CI)	Prob (95% CI)	p-value
NRM	NGS MRD *IDH1* neg n=95	NGS MRD *IDH1* pos n=53	0.709
3 mo	2(0-5)%	4(0-9)%	
6 mo	5(1-10)%	9(1-17)%	
1 yr	9(4-15)%	11(3-20)%	
2 yr	11(4-18)%	11(3-20)%	
3 yr	11(4-18)%	15(4-26)%	
Relapse	NGS MRD *IDH1* neg n=95	NGS MRD *IDH1* pos n=53	0.459
3 mo	5(1-10)%	4(0-9)%	
6 mo	11(4-17)%	17(7-27)%	
1 yr	17(9-24)%	23(11-34)%	
2 yr	20(11-28)%	26(13-38)%	
3 yr	28(16-40)%	26(13-38)%	
RFS	NGS MRD *IDH1* neg n=95	NGS MRD *IDH1* pos n=53	0.336
3 mo	93(85-96)%	92(81-97)%	
6 mo	84(75-90)%	74(60-83)%	
1 yr	74(64-81)%	66(52-77)%	
2 yr	69(59-78)%	63(48-75)%	
3 yr	61(48-72)%	60(44-72)%	
OS	NGS MRD *IDH1* neg n=95	NGS MRD *IDH1* pos n=53	0.391
3 mo	98(92-99)%	96(86-99)%	
6 mo	92(84-96)%	87(74-93)%	
1 yr	84(75-90)%	79(66-88)%	
2 yr	75(65-83)%	69(53-80)%	
3 yr	67(54-78)%	65(49-78)%	

B

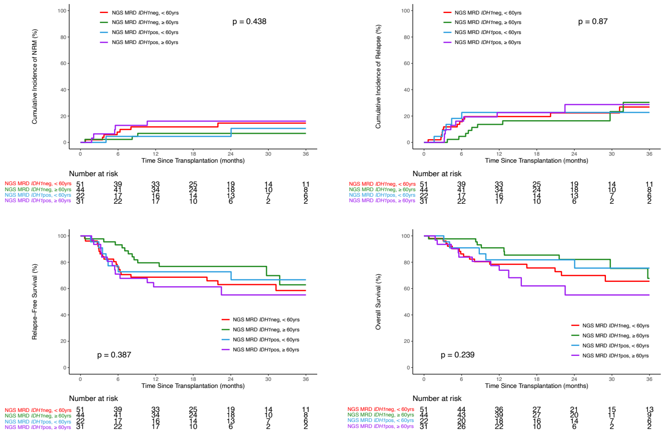

NGS MRD *IDH1* and Age Group

Outcomes	Prob (95% CI)	Prob (95% CI)	Prob (95% CI)	Prob (95% CI)	p-value
NRM	NGS MRD *IDH1* neg <60yrs: n=51	NGS MRD *IDH1* neg ≥60yrs: n=44	NGS MRD *IDH1* pos <60yrs: n=22	NGS MRD *IDH1* pos ≥60yrs: n=31	0.438
3 mo	2(0-6)%	2(0-7)%	0(0-0)%	6(0-15)%	
6 mo	8(0-15)%	2(0-7)%	5(0-13)%	13(1-25)%	
1 yr	12(3-21)%	7(0-14)%	5(0-13)%	16(3-29)%	
2 yr	15(4-25)%	7(0-14)%	5(0-13)%	16(3-29)%	
3 yr	15(4-25)%	7(0-14)%	11(0-25)%	16(3-29)%	
Relapse	NGS MRD *IDH1* neg <60yrs: n=51	NGS MRD *IDH1* neg ≥60yrs: n=44	NGS MRD *IDH1* pos <60yrs: n=22	NGS MRD *IDH1* pos ≥60yrs: n=31	0.87
3 mo	10(2-18)%	0(0-0)%	9(0-21)%	0(0-0)%	
6 mo	16(6-26)%	5(0-11)%	18(2-35)%	16(3-29)%	
1 yr	20(9-31)%	14(3-24)%	23(5-41)%	23(8-38)%	
2 yr	22(10-34)%	16(5-28)%	23(5-41)%	29(10-47)%	
3 yr	27(13-41)%	30(10-51)%	23(5-41)%	29(10-47)%	
RFS	NGS MRD *IDH1* neg <60yrs: n=51	NGS MRD *IDH1* neg ≥60yrs: n=44	NGS MRD *IDH1* pos <60yrs: n=22	NGS MRD *IDH1* pos ≥60yrs: n=31	0.387
3 mo	88(76-95)%	98(85-100)%	91(68-98)%	94(77-98)%	
6 mo	76(62-86)%	93(80-98)%	77(54-90)%	71(52-84)%	
1 yr	69(54-79)%	80(64-89)%	73(49-87)%	61(42-76)%	
2 yr	63(48-75)%	77(61-87)%	73(49-87)%	55(34-72)%	
3 yr	59(42-72)%	63(39-79)%	67(42-83)%	55(34-72)%	
OS	NGS MRD *IDH1* neg <60yrs: n=51	NGS MRD *IDH1* neg ≥60yrs: n=44	NGS MRD *IDH1* pos <60yrs: n=22	NGS MRD *IDH1* pos ≥60yrs: n=31	0.239
3 mo	98(87-100)%	98(85-100)%	100%	94(77-98)%	
6 mo	86(73-93)%	98(85-100)%	91(68-98)%	84(66-93)%	
1 yr	78(64-87)%	91(78-96)%	82(59-93)%	77(58-89)%	
2 yr	70(54-81)%	82(66-91)%	82(59-93)%	55(31-74)%	
3 yr	66(48-78)%	68(43-84)%	76(50-89)%	55(31-74)%	

C

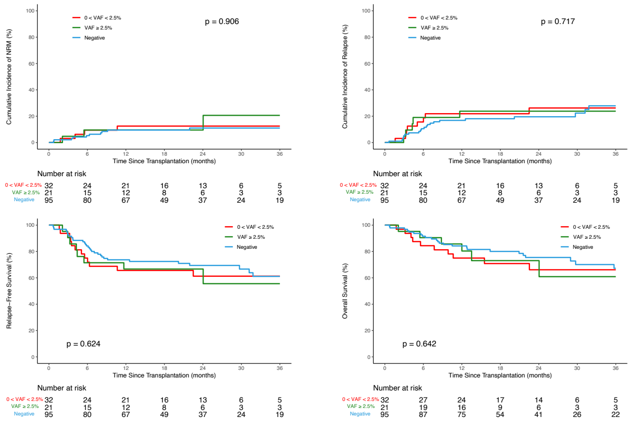

VAF Groups

Outcomes	Prob (95% CI)	Prob (95% CI)	Prob (95% CI)	p-value
NRM	0<VAF<2.5% =32	VAF ≤ 2.5% =21	Negative=95	0.906
3 mo	3(0-9)%	5(0-14)%	2(0-5)%	
6 mo	9(0-20)%	10(0-22)%	5(1-10)%	
1 yr	12(1-24)%	10(0-22)%	9(4-15)%	
2 yr	12(1-24)%	10(0-22)%	11(4-18)%	
3 yr	12(1-24)%	21(0-45)%	11(4-18)%	
Relapse	0<VAF<2.5% =32	VAF ≤ 2.5% =21	Negative=95	0.717
3 mo	3(0-9)%	5(0-14)%	5(1-10)%	
6 mo	16(3-28)%	19(2-36)%	11(4-17)%	
1 yr	22(7-36)%	24(5-43)%	17(9-24)%	
2 yr	26(10-42)%	24(5-43)%	20(11-28)%	
3 yr	26(10-42)%	24(5-43)%	28(16-40)%	
RFS	0<VAF<2.5% =32	VAF ≤ 2.5% =21	Negative=95	0.624
3 mo	94(77-98)%	90(67-98)%	93(85-96)%	
6 mo	75(56-87)%	71(47-86)%	84(75-90)%	
1 yr	66(47-79)%	67(43-83)%	74(64-81)%	
2 yr	61(42-76)%	67(43-83)%	69(59-78)%	
3 yr	61(42-76)%	56(27-77)%	61(48-72)%	
OS	0<VAF<2.5% =32	VAF ≤ 2.5% =21	Negative=95	0.642
3 mo	97(80-100)%	95(71-99)%	98(92-99)%	
6 mo	84(66-93)%	90(67-98)%	92(84-96)%	
1 yr	75(56-87)%	86(62-95)%	84(75-90)%	
2 yr	66(46-80)%	73(46-88)%	75(65-83)%	
3 yr	66(46-80)%	61(29-82)%	67(54-78)%	

Supplementary Figure 5. NGS MRD status for *IDH1*-mutated AML patients and the association with clinical outcomes after allogeneic hematopoietic cell transplant stratified by baseline mutation groups. Cumulative incidence of non-relapse mortality (NRM, top left) and relapse (top right), relapse-free survival (RFS, bottom left) and overall survival (OS, bottom right) shown at 24 months based on next generation sequencing measurable residual disease (NGS MRD) status and baseline patient mutation groups (A) *IDH1*-mutated AML patients with either *NPM1* and/or *FLT3*-ITD mutations at baseline (n=69) and (B) *IDH1*-mutated AML patients without *NPM1* or *FLT3*-ITD mutations at baseline (n=79). Point estimates at different time points are shown in the table (far right). Overall P values: Gray's test for non-relapse mortality (NRM) and relapse; log-rank test for relapse-free survival (RFS) and overall survival (OS). P values for pointwise estimations at different time points: z-test. Confidence interval, CI; Probability, prob; Month, mo; Year, yr; pos, positive; neg, negative.

A

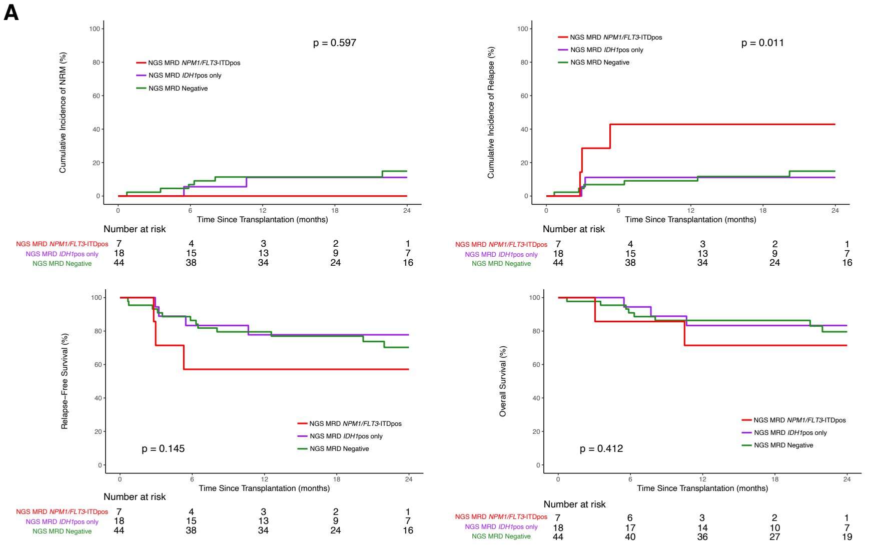

***NPM1/FLT3*-ITD Baseline Patients NGS MRD Status**

Outcomes	Prob (95% CI)	Prob (95% CI)	Prob (95% CI)	p-value
NRM	*NPM1/FLT3*-ITDpos=7	*IDH1*pos only=18	MRD Negative=44	0.597
3 mo	0(0-0)%	0(0-0)%	2(0-7)%	
6 mo	0(0-0)%	6(0-16)%	7(0-14)%	
1 yr	0(0-0)%	11(0-26)%	11(2-21)%	
2 yr	0(0-0)%	11(0-26)%	15(3-26)%	
Relapse	*NPM1/FLT3*-ITDpos=7	*IDH1*pos only=18	MRD Negative=44	0.011
3 mo	29(0-65)%	6(0-16)%	5(0-11)%	
6 mo	43(2-83)%	11(0-26)%	7(0-14)%	
1 yr	43(2-83)%	11(0-26)%	9(0-18)%	
2 yr	43(2-83)%	11(0-26)%	15(4-26)%	
RFS	*NPM1/FLT3*-ITDpos=7	*IDH1*pos only=18	MRD Negative=44	0.145
3 mo	71(26-92)%	94(67-99)%	93(80-98)%	
6 mo	57(17-84)%	83(57-94)%	86(72-94)%	
1 yr	57(17-84)%	78(51-91)%	80(64-89)%	
2 yr	57(17-84)%	78(51-91)%	70(53-82)%	
OS	*NPM1/FLT3*-ITDpos=7	*IDH1*pos only=18	MRD Negative=44	0.412
3 mo	100%	100%	98(85-100)%	
6 mo	86(33-98)%	94(67-99)%	91(78-96)%	
1 yr	71(26-92)%	83(57-94)%	86(72-94)%	
2 yr	71(26-92)%	83(57-94)%	80(63-89)%	

B

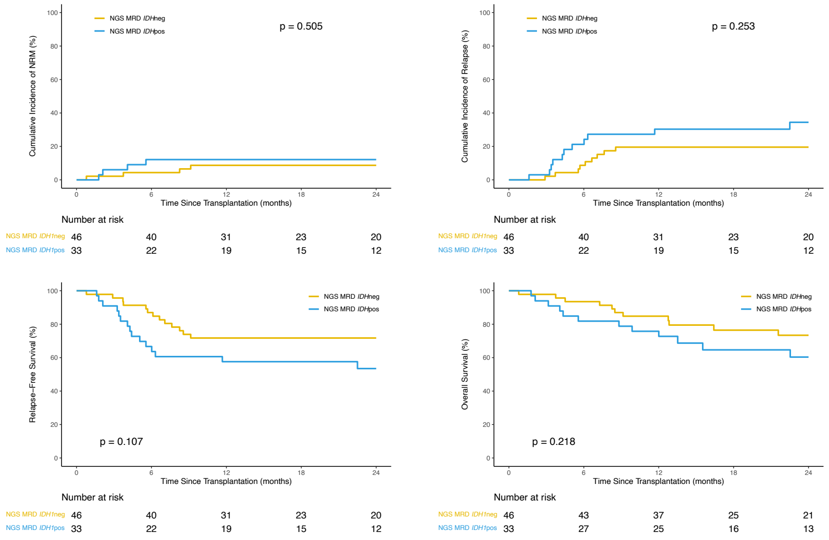

***IDH1* Only Baseline Patients (without *NPM1* or *FLT3*-ITD) NGS MRD Status**

Outcomes	Prob (95% CI)	Prob (95% CI)	p-value
NRM	NGS MRD *IDH*neg=46	NGS MRD *IDH*pos=33	0.505
3 mo	2(0-6)%	6(0-14)%	
6 mo	4(0-10)%	12(1-23)%	
1 yr	9(0-17)%	12(1-23)%	
2 yr	9(0-17)%	12(1-23)%	
3 yr	9(0-17)%	17(3-31)%	
Relapse	NGS MRD *IDH*neg=46	NGS MRD *IDH*pos=33	0.253
3 mo	2(0-6)%	3(0-9)%	
6 mo	9(0-17)%	21(7-35)%	
1 yr	20(8-31)%	30(14-46)%	
2 yr	20(8-31)%	34(17-52)%	
3 yr	29(13-46)%	34(17-52)%	
RFS	NGS MRD *IDH*neg=46	NGS MRD *IDH*pos=33	0.107
3 mo	96(84-99)%	91(74-97)%	
6 mo	87(73-94)%	67(48-80)%	
1 yr	72(56-82)%	58(39-72)%	
2 yr	72(56-82)%	53(35-69)%	
3 yr	62(43-76)%	49(30-65)%	
OS	NGS MRD *IDH*neg=46	NGS MRD *IDH*pos=33	0.218
3 mo	98(86-100)%	94(78-98)%	
6 mo	93(81-98)%	82(64-91)%	
1 yr	85(71-92)%	76(57-87)%	
2 yr	73(57-84)%	60(40-76)%	
3 yr	68(50-81)%	56(35-72)%	

Supplementary Figure 6. NGS MRD status for *IDH1*-mutated AML patients and the association with clinical outcomes after allogeneic hematopoietic cell transplant stratified by conditioning intensity. Cumulative incidence of non-relapse mortality (NRM, top left) and relapse (top right), relapse-free survival (RFS, bottom left) and overall survival (OS, bottom right) shown at 36 months based on next generation sequencing measurable residual disease (NGS MRD) *IDH1* status and conditioning intensity. Point estimates at different time points are shown in the table (far right). Overall P values: Gray's test for non-relapse mortality (NRM) and relapse; log-rank test for relapse-free survival (RFS) and overall survival (OS). P values for pointwise estimations at different time points: z-test. Confidence interval, CI; Probability, prob; Month, mo; Year, yr; pos, positive; neg, negative.

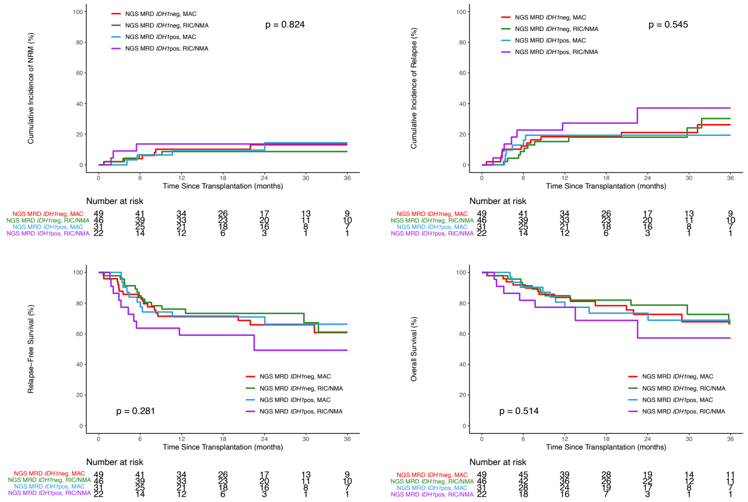

NGS MRD *IDH1* Status and Conditioning Intensity

Outcomes	Prob (95% CI)	Prob (95% CI)	Prob (95% CI)	Prob (95% CI)	p-value
NRM	NGS MRD *IDH1* neg, MAC, n=49	NGS MRD *IDH1* neg, RIC/NMA, n=46	NGS MRD *IDH1* pos, MAC, n=31	NGS MRD *IDH1* pos, RIC/NMA, n=22	0.824
3 mo	2(0-6)%	2(0-6)%	0(0-0)%	9(0-21)%	
6 mo	4(0-10)%	7(0-14)%	6(0-15)%	14(0-28)%	
1 yr	10(2-19)%	9(0-17)%	10(0-20)%	14(0-28)%	
2 yr	13(3-23)%	9(0-17)%	10(0-20)%	14(0-28)%	
3 yr	13(3-23)%	9(0-17)%	14(1-28)%	14(0-28)%	
Relapse	NGS MRD *IDH1* neg, MAC, n=49	NGS MRD *IDH1* neg, RIC/NMA, n=46	NGS MRD *IDH1* pos, MAC, n=31	NGS MRD *IDH1* pos, RIC/NMA, n=22	0.545
3 mo	10(2-19)%	0(0-0)%	0(0-0)%	9(0-21)%	
6 mo	12(3-22)%	9(0-17)%	13(1-25)%	23(5-41)%	
1 yr	18(7-29)%	15(5-26)%	19(5-34)%	27(8-46)%	
2 yr	21(9-33)%	18(6-30)%	19(5-34)%	37(12-63)%	
3 yr	26(11-41)%	30(11-49)%	19(5-34)%	37(12-63)%	
RFS	NGS MRD *IDH1* neg, MAC, n=49	NGS MRD *IDH1* neg, RIC/NMA, n=46	NGS MRD *IDH1* pos, MAC, n=31	NGS MRD *IDH1* pos, RIC/NMA, n=22	0.281
3 mo	88(75-94)%	98(86-100)%	100%	82(59-93)%	
6 mo	84(70-91)%	85(71-92)%	81(62-91)%	64(40-80)%	
1 yr	71(57-82)%	76(61-86)%	71(52-84)%	59(36-76)%	
2 yr	66(50-78)%	73(58-84)%	71(52-84)%	49(24-70)%	
3 yr	61(43-75)%	61(40-77)%	66(46-80)%	49(24-70)%	
OS	NGS MRD *IDH1* neg, MAC, n=49	NGS MRD *IDH1* neg, RIC/NMA, n=46	NGS MRD *IDH1* pos, MAC, n=31	NGS MRD *IDH1* pos, RIC/NMA, n=22	0.514
3 mo	98(86-100)%	98(86-100)%	100%	91(68-98)%	
6 mo	92(80-97)%	91(78-97)%	90(73-97)%	82(59-93)%	
1 yr	84(70-91)%	85(71-92)%	81(62-91)%	77(54-90)%	
2 yr	73(56-84)%	79(63-88)%	73(54-86)%	57(27-79)%	
3 yr	68(50-81)%	67(44-82)%	69(48-83)%	57(27-79)%	

Supplementary Figure 7. Multivariable regression analysis for the rate of relapse of *IDH1*-mutated AML patients. Models were selected stepwise using likelihood ratio test with covariates including *IDH1* next-generation sequencing measurable residual disease (NGS-MRD) status and baseline characteristics including age, sex, race, hematopoietic cell transplant specific comorbidity index, Karnofsky performance status, AML type, ELN risk group, baseline mutation groups, conditioning regimen, graft type, donor group, antithymocyte globulin usage. Baseline groups for the reported hazard ratio are favorable for ELN, and negative for flow MRD.

Multivariable Regression for Relapse

	hazard ratio	p−value
ELN: Adverse	4.088 (0.919−18.19)	0.065
ELN: Intermediate	4.809 (1.118−20.692)	0.035
Flow MRD: Positive	2.658 (1.052−6.719)	0.039

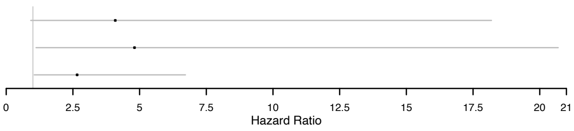

Supplementary Figure 8. Patient transplant years for *IDH1*-mutated AML patients and the association with clinical outcomes after allogeneic hematopoietic cell transplant stratified by NGS MRD status. Cumulative incidence of non-relapse mortality (NRM, top left) and relapse (top right), relapse-free survival (RFS, bottom left) and overall survival (OS, bottom right) shown at 36 months based on *IDH1* next generation sequencing measurable residual disease (NGS MRD) status based on year of transplantation (before or after July 2018) for (A) all patients and (B) patients stratified by *IDH1* NGS MRD status. Point estimates at different time points are shown in the table (far right). Overall P values: Gray's test for non-relapse mortality (NRM) and relapse; log-rank test for relapse-free survival (RFS) and overall survival (OS). P values for pointwise estimations at different time points: z-test. Confidence interval, CI; Probability, prob; Month, mo; Year, yr; pos, positive; neg, negative.

A

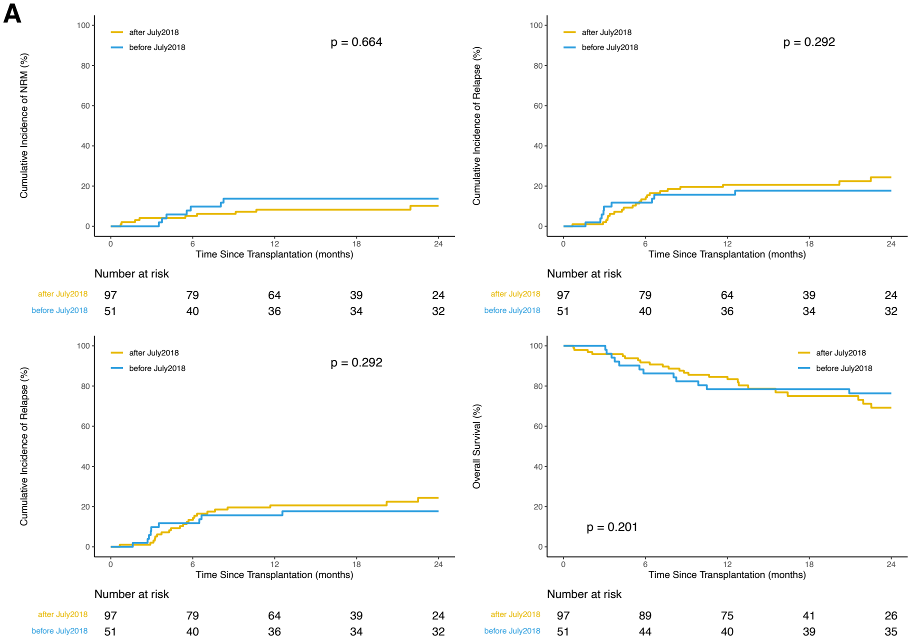

Patient Transplant Year

Outcomes	Prob (95% CI)	Prob (95% CI)	p-value
NRM	after July2018=97	before July2018=51	0.664
3 mo	4(0-8)%	0(0-0)%	
6 mo	5(1-10)%	10(2-18)%	
1 yr	8(3-14)%	14(4-23)%	
2 yr	10(4-17)%	14(4-23)%	
3 yr	13(5-21)%	14(4-23)%	
Relapse	after July2018=97	before July2018=51	0.292
3 mo	2(0-5)%	10(2-18)%	
6 mo	13(7-20)%	12(3-21)%	
1 yr	21(13-29)%	16(6-26)%	
2 yr	24(15-34)%	18(7-28)%	
3 yr	56(0-100)%	22(10-34)%	
RFS	after July2018=97	before July2018=51	0.558
3 mo	94(87-97)%	90(78-96)%	
6 mo	81(72-88)%	78(64-87)%	
1 yr	71(61-79)%	71(56-81)%	
2 yr	65(54-75)%	69(54-79)%	
3 yr	31(2-71)%	64(49-76)%	
OS	after July2018=97	before July2018=51	0.201
3 mo	96(89-98)%	100%	
6 mo	92(84-96)%	86(73-93)%	
1 yr	85(76-90)%	78(64-87)%	
2 yr	69(57-79)%	76(62-86)%	
3 yr	22(1-60)%	74(59-84)%	

B

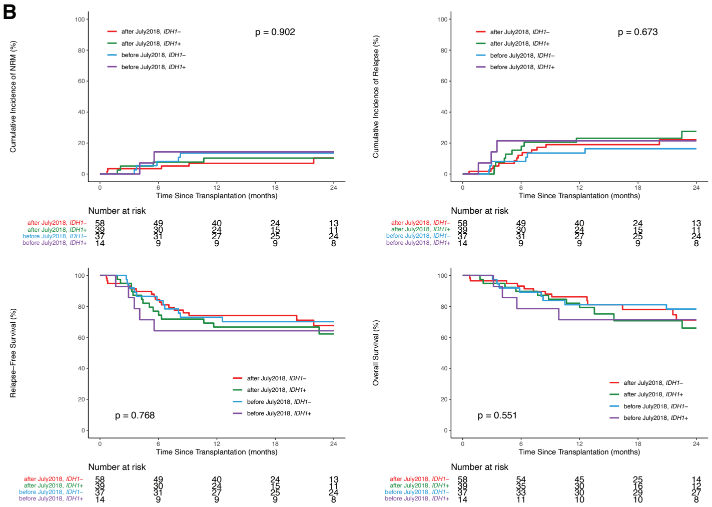

Patient Transplant Year and NGS MRD *IDH1* Status

Outcomes	Prob (95% CI)	Prob (95% CI)	Prob (95% CI)	Prob (95% CI)	p-value
NRM	after July2018, *IDH1*-=58	after July2018, *IDH1*+=39	before July2018, *IDH1*-=37	before July2018, *IDH1*+=14	0.902
3 mo	3(0-8)%	5(0-12)%	0(0-0)%	0(0-0)%	
6 mo	3(0-8)%	8(0-16)%	8(0-17)%	14(0-34)%	
1 yr	7(0-13)%	10(1-20)%	14(2-25)%	14(0-34)%	
2 yr	10(1-19)%	10(1-20)%	14(2-25)%	14(0-34)%	
3 yr	10(1-19)%	16(1-32)%	14(2-25)%	14(0-34)%	
Relapse	after July2018, *IDH1*-=58	after July2018, *IDH1*+=39	before July2018, *IDH1*-=37	before July2018, *IDH1*+=14	0.673
3 mo	3(0-8)%	0(0-0)%	8(0-17)%	14(0-33)%	
6 mo	12(4-21)%	15(4-27)%	8(0-17)%	21(0-44)%	
1 yr	19(9-29)%	23(10-37)%	14(2-25)%	21(0-44)%	
2 yr	22(11-34)%	28(12-43)%	16(4-28)%	21(0-44)%	
3 yr	90(0-100)%	28(12-43)%	22(8-36)%	21(0-44)%	
RFS	after July2018, *IDH1*-=58	after July2018, *IDH1*+=39	before July2018, *IDH1*-=37	before July2018, *IDH1*+=14	0.768
3 mo	93(83-97)%	95(81-99)%	92(77-97)%	86(54-96)%	
6 mo	84(72-92)%	77(60-87)%	84(67-92)%	64(34-83)%	
1 yr	74(61-84)%	67(50-79)%	73(56-84)%	64(34-83)%	
2 yr	68(52-79)%	62(44-76)%	70(53-82)%	64(34-83)%	
3 yr	0(NA-NA)%	56(36-72)%	64(47-78)%	64(34-83)%	
OS	after July2018, *IDH1*-=58	after July2018, *IDH1*+=39	before July2018, *IDH1*-=37	before July2018, *IDH1*+=14	0.551
3 mo	97(87-99)%	95(81-99)%	100%	100%	
6 mo	93(83-97)%	90(75-96)%	89(74-96)%	79(47-93)%	
1 yr	86(74-93)%	82(66-91)%	81(64-90)%	71(41-88)%	
2 yr	71(54-83)%	66(46-80)%	78(61-88)%	71(41-88)%	
3 yr	0(NA-NA)%	60(38-76)%	75(58-86)%	71(41-88)%	

Supplementary Table 1. Variants detected by next-generation sequencing in the blood of *IDH1* mutated AML patients prior to transplant conditioning.

Subject ID	Gene	HGVSp	HGVSc	VAF	Alternate Observations	Deep Alternate Observations	Unique Alternate Observations	Depth	Deep Depth	Variant Consequence	Chromosome	Position	Reference	Alternate	Previously reported*	ddPCR validated	ddPCR VAF
CH 012119	NPM1	NP_002511.1:p.Trp288CysfsTer12	NM_002520.6:c.860_863dup	0.0001	4	1	1	37243	10054	frameshift	5	170837543	C	CTCTG	Yes	Yes	0.00015
CH 012020	IDH1	NP_005887.2:p.Arg132Cys	NM_005896.3:c.394C>T	0.006	280	118	117	36952	19630	missense	2	209113113	G	A	Yes	Yes	0.0087184
CH 012505	IDH1	NP_005887.2:p.Arg132Ser	NM_005896.3:c.394C>A	0.2104	6529	3600	562	30474	17113	missense	2	209113113	G	T	Yes	Yes	0.21690
CH 012247	IDH1	NP_005887.2:p.Arg132His	NM_005896.3:c.395G>A	0.0124	572	170	207	40301	13742	missense	2	209113112	C	T	Yes	Yes	0.01460
CH 012139	IDH1	NP_005887.2:p.Arg132Cys	NM_005896.3:c.394C>T	0.0142	496	203	179	28504	14249	missense	2	209113113	G	A	Yes	Yes	0.01799
CH 012442	IDH1	NP_005887.2:p.Arg132His	NM_005896.3:c.395G>A	0.0082	438	185	168	50814	22624	missense	2	209113112	C	T	Yes	Yes	0.00942
CH 012476	IDH1	NP_005887.2:p.Arg132Gly	NM_005896.3:c.394C>G	0.3379	12299	3240	700	35090	9589	missense	2	209113113	G	C	Yes	Yes	0.34389
CH 012177	IDH1	NP_005887.2:p.Arg132Cys	NM_005896.3:c.394C>T	0.0627	2896	1236	429	40875	19728	missense	2	209113113	G	A	Yes	Yes	0.06833
CH 012228	IDH1	NP_005887.2:p.Arg132Cys	NM_005896.3:c.394C>T	0.0042	187	74	83	34772	17459	missense	2	209113113	G	A	Yes	Yes	0.00407
CH 016941	FLT3	NP_004110.2:p.Glu598_Tyr599ins PheAspPheArgGluTyrGlu	NM_004119.2:c.1775_1795dup	0.0002	17		16	74735	N/A	FLT3-ITD	13	28608260	T	TATTCATATTCTC TGAAATCAA	Yes	Not Done	Not Done
CH 016498	IDH1	NP_005887.2:p.Arg132Ser	NM_005896.3:c.394C>A	0.0011	65	23	42	44701	21453	missense	2	209113113	G	T	Yes	Yes	0.00090
CH 016870	IDH1	NP_005887.2:p.Arg132Gly	NM_005896.3:c.394C>G	0.0106	298	166	133	26660	15691	missense	2	209113113	G	C	Yes	Yes	0.01208
CH 016683	IDH1	NP_005887.2:p.Arg132Ser	NM_005896.3:c.394C>A	0.0103	239	147	118	23713	14224	missense	2	209113113	G	T	Yes	Yes	0.00980
CH 016673	IDH1	NP_005887.2:p.Arg132Gly	NM_005896.3:c.394C>G	0.0195	600	345	221	30821	17734	missense	2	209113113	G	C	Yes	Yes	0.02029
CH 016649	IDH1	NP_005887.2:p.Arg132Cys	NM_005896.3:c.394C>T	0.0223	619	342	220	25060	15306	missense	2	209113113	G	A	Yes	Yes	0.02783
CH 016764	IDH1	NP_005887.2:p.Arg132His	NM_005896.3:c.395G>A	0.0022	83	37	52	28437	17182	missense	2	209113112	C	T	Yes	Yes	0.00320
CH 016546	FLT3	NP_004110.2:p.Arg595_Leu601dup	NM_004119.2:c.1784_1804dup	0.0004	30		17	84776	N/A	FLT3-ITD	13	28608251	T	TTGAGATCATAT TCATATTCTC	Yes	Not Done	Not Done
CH 016546	NPM1	NP_002511.1:p.Trp288CysfsTer12	NM_002520.6:c.860_863dup	0.0009	31	4	20	33577	10783	frameshift	5	170837543	C	CTCTG	Yes	Yes	0.00087
CH 016548	NPM1	NP_002511.1:p.Trp288CysfsTer12	NM_002520.6:c.860_863dup	0.0001	3	0	1	22233	8099	frameshift	5	170837543	C	CTCTG	Yes	Yes	0.00023
CH 016989	IDH1	NP_005887.2:p.Arg132Cys	NM_005896.3:c.394C>T	0.0292	1011	575	294	32448	19666	missense	2	209113113	G	A	Yes	Yes	0.03584
CH 016748	IDH1	NP_005887.2:p.Arg132Cys	NM_005896.3:c.394C>T	0.0355	1232	729	313	32558	20552	missense	2	209113113	G	A	Yes	Yes	0.03998
CH 016748	NPM1	NP_002511.1:p.Trp288CysfsTer12	NM_002520.6:c.860_863dup	0.0005	17	3	10	34071	15101	frameshift	5	170837543	C	CTCTG	Yes	Yes	0.00019
CH 016486	IDH1	NP_005887.2:p.Arg132Cys	NM_005896.3:c.394C>T	0.0021	46	28	35	20823	13423	missense	2	209113113	G	A	Yes	Yes	0.00329
CH 016466	IDH1	NP_005887.2:p.Arg132Cys	NM_005896.3:c.394C>T	0.0026	84	54	54	32440	20414	missense	2	209113113	G	A	Yes	Yes	0.00252
CH 016799	IDH1	NP_005887.2:p.Arg132Gly	NM_005896.3:c.394C>G	0.0019	54	25	41	23572	13146	missense	2	209113113	G	C	Yes	Yes	0.00150
CH 017017	IDH1	NP_005887.2:p.Arg132Cys	NM_005896.3:c.394C>T	0.0022	62	35	45	25384	15728	missense	2	209113113	G	A	Yes	Yes	0.00263
CH 016447	IDH1	NP_005887.2:p.Arg132Cys	NM_005896.3:c.394C>T	0.0268	1085	608	293	37934	22717	missense	2	209113113	G	A	Yes	Yes	0.02671
CH 016484	IDH1	NP_005887.2:p.Arg132Cys	NM_005896.3:c.394C>T	0.0056	203	117	97	33383	20940	missense	2	209113113	G	A	Yes	Yes	0.00610
CH 016589	IDH1	NP_005887.2:p.Arg132Cys	NM_005896.3:c.394C>T	0.0025	105	50	60	32822	19978	missense	2	209113113	G	A	Yes	Yes	0.00343
CH 016995	IDH1	NP_005887.2:p.Arg132Cys	NM_005896.3:c.394C>T	0.0272	904	536	236	32960	19677	missense	2	209113113	G	A	Yes	Yes	0.02704
CH 016829	IDH1	NP_005887.2:p.Arg132Ser	NM_005896.3:c.394C>A	0.0011	124	20	76	51081	17490	missense	2	209113113	G	T	Yes	Yes	0.00229
CH 016825	IDH1	NP_005887.2:p.Arg132His	NM_005896.3:c.395G>A	0.3064	14168	7169	872	44813	23394	missense	2	209113112	C	T	Yes	Yes	0.31749
CH 016969	IDH1	NP_005887.2:p.Arg132Cys	NM_005896.3:c.394C>T	0.0099	555	237	200	47267	23840	missense	2	209113113	G	A	Yes	Yes	0.01298
CH 017043	IDH1	NP_005887.2:p.Arg132Cys	NM_005896.3:c.394C>T	0.0066	275	135	136	36039	20601	missense	2	209113113	G	A	Yes	Yes	0.00789
CH 017073	IDH1	NP_005887.2:p.Arg132Cys	NM_005896.3:c.394C>T	0.0423	1356	764	330	30626	18074	missense	2	209113113	G	A	Yes	Yes	0.04679
CH 016893	IDH1	NP_005887.2:p.Arg132Cys	NM_005896.3:c.394C>T	0.0826	4273	1703	568	44684	20612	missense	2	209113113	G	A	Yes	Yes	0.10241
CH 016946	IDH1	NP_005887.2:p.Arg132Cys	NM_005896.3:c.394C>T	0.0336	1023	511	275	26841	15202	missense	2	209113113	G	A	Yes	Yes	0.03914
CH 016958	IDH1	NP_005887.2:p.Arg132Gly	NM_005896.3:c.394C>G	0.3531	6344	3132	534	18124	8870	missense	2	209113113	G	C	Yes	Yes	0.35364
CH 017008	IDH1	NP_005887.2:p.Arg132His	NM_005896.3:c.395G>A	0.0165	587	253	200	34690	15290	missense	2	209113112	C	T	Yes	Yes	0.01861
CH 017078	IDH1	NP_005887.2:p.Arg132Cys	NM_005896.3:c.394C>T	0.0024	120	42	56	33768	17434	missense	2	209113113	G	A	Yes	Yes	0.00373
CH 017022	IDH1	NP_005887.2:p.Arg132His	NM_005896.3:c.395G>A	0.4846	25024	7806	884	51843	16108	missense	2	209113112	C	T	Yes	Yes	0.48050
CH 017053	IDH1	NP_005887.2:p.Arg132Gly	NM_005896.3:c.394C>G	0.001	31	17	22	34789	16631	missense	2	209113113	G	C	Yes	Yes	0.00050
CH 017091	IDH1	NP_005887.2:p.Arg132Gly	NM_005896.3:c.394C>G	0.0121	479	224	158	35214	18509	missense	2	209113113	G	C	Yes	Yes	0.00598
CH 017495	IDH1	NP_005887.2:p.Arg132Gly	NM_005896.3:c.394C>G	0.1225	5616	1950	555	43459	15916	missense	2	209113113	G	C	Yes	Yes	0.12944
CH 017385	IDH1	NP_005887.2:p.Arg132His	NM_005896.3:c.395G>A	0.0224	1114	363	301	40319	16232	missense	2	209113112	C	T	No	Yes	0.031
CH 017402	IDH1	NP_005887.2:p.Arg132His	NM_005896.3:c.395G>A	0.0352	1349	445	353	34420	12628	missense	2	209113112	C	T	No	Yes	0.0424
CH 017402	NPM1	NP_002511.1:p.Trp288CysfsTer12	NM_002520.6:c.863_864insCTTG	0.0386	1035	143	317	26807	3750	frameshift	5	170837545	C	CTGCT	No	Not Done	Not Done
CH 017425	FLT3	NP_004110.2:p.Gly583_Leu601dup	NM_004119.2:c.1747_1803dup	0.0118	576		63	48885	N/A	FLT3-ITD	13	28608253	G	GAGATCATATTC ATATTCTCTGAA ATCAACGTAGAA GTACTCATTATC TGAGGAGCCG	No	Not Done	Not Done
CH 017425	NPM1	NP_002511.1:p.Trp288CysfsTer12	NM_002520.6:c.860_863dup	0.0014	24	4	17	16781	2673	frameshift	5	170837543	C	CTCTG	No	Not Done	Not Done
CH 017430	IDH1	NP_005887.2:p.Arg132Cys	NM_005896.3:c.393_394delinsCT	0.0148	630	166	215	39016	11222	missense	2	209113113	GA	AG	No	Not Done	Not Done
CH 017444	IDH1	NP_005887.2:p.Arg132Cys	NM_005896.3:c.394C>T	0.0028	154	43	81	38875	15569	missense	2	209113113	G	A	No	Yes	0.0047
CH 017474	IDH1	NP_005887.2:p.Arg132Cys	NM_005896.3:c.394C>T	0.0147	481	105	158	33924	7148	missense	2	209113113	G	A	No	Yes	0.0143
CH 017505	IDH1	NP_005887.2:p.Arg132Cys	NM_005896.3:c.394C>T	0.0255	900	429	276	33139	16820	missense	2	209113113	G	A	No	Yes	0.0273
CH 017549	IDH1	NP_005887.2:p.Arg132Cys	NM_005896.3:c.394C>T	0.0064	195	59	97	25964	9216	missense	2	209113113	G	A	No	Yes	0.008
CH 017580	IDH1	NP_005887.2:p.Arg132Cys	NM_005896.3:c.394C>T	0.0101	343	58	140	32296	5740	missense	2	209113113	G	A	No	Yes	0.0109
CH 017589	IDH1	NP_005887.2:p.Arg132Cys	NM_005896.3:c.394C>T	0.1193	3316	1242	485	26777	10414	missense	2	209113113	G	A	No	Yes	0.1257
CH 017592	IDH1	NP_005887.2:p.Arg132Leu	NM_005896.3:c.395G>T	0.1298	4953	1451	531	35798	11180	missense	2	209113112	C	A	No	Not Done	Not Done
CH 017607	IDH1	NP_005887.2:p.Arg132Cys	NM_005896.3:c.394C>T	0.0566	1518	904	354	25707	15960	missense	2	209113113	G	A	No	Yes	0.0586
CH 017634	IDH1	NP_005887.2:p.Arg132His	NM_005896.3:c.395G>A	0.4115	10535	5534	655	25471	13449	missense	2	209113112	C	T	No	Yes	0.4048
CH 017640	IDH1	NP_005887.2:p.Arg132Cys	NM_005896.3:c.394C>T	0.0094	466	132	183	39332	13980	missense	2	209113113	G	A	No	Yes	0.0121
CH 017643	IDH1	NP_005887.2:p.Arg132Gly	NM_005896.3:c.394C>G	0.0352	1572	462	384	42114	13117	missense	2	209113113	G	C	No	Yes	0.037
CH 017645	IDH1	NP_005887.2:p.Arg132His	NM_005896.3:c.395G>A	0.001	79	17	53	40408	17558	missense	2	209113112	C	T	No	Yes	0.0018
